# Preoperative Indicators of Graft Rejection in Patients With Heart Transplantation: A Single-Center Retrospective Study

**DOI:** 10.7759/cureus.97730

**Published:** 2025-11-25

**Authors:** Laurentiu Huma, Horatiu Suciu, Diana Andreea Moldovan, Radu-Adrian Suteu, Sergiu Morosanu, Dragos-Florin Baba, Anca-Ileana Sin

**Affiliations:** 1 Department of Doctoral Studies, George Emil Palade University of Medicine, Pharmacy, Science and Technology of Târgu Mureș, Târgu Mureș, ROU; 2 Department of Cell and Molecular Biology, George Emil Palade University of Medicine, Pharmacy, Science and Technology of Târgu Mureș, Târgu Mureș, ROU; 3 Department of Cardiology I, Emergency Institute for Cardiovascular Diseases and Transplantation of Târgu Mureș, Târgu Mureș, ROU; 4 Department of Surgery, George Emil Palade University of Medicine, Pharmacy, Science and Technology of Târgu Mureş, Târgu Mureș, ROU; 5 Department of Cardiovascular Surgery, Emergency Institute for Cardiovascular Diseases and Transplantation of Târgu Mureş, Târgu Mureș, ROU; 6 Department of Family Medicine, George Emil Palade University of Medicine, Pharmacy, Science and Technology of Târgu Mureș, Târgu Mureș, ROU

**Keywords:** cardiomyopathy, graft rejection, heart failure, quilty effect, transplant

## Abstract

Introduction: Heart failure (HF) represents a syndrome characterized by the heart’s inability to fulfill its pump role due to a decrease in its mechanical function or abnormal filling pressures. This disease is one of the focus areas in modern research, with medication and mechanical assistance devices being constantly developed. The final treatment option, when all the medical and device therapies have been exhausted, is represented by the heart transplant. Patients undergoing heart transplant face biological and psychological challenges, which must be addressed by attending physicians to increase the survival and quality of life of these individuals. The aim of this study was to describe the population who received a heart transplant in our center over 12 years and to assess potential connections between the etiology of cardiomyopathy associated with end-stage HF of the organ recipient and clinical and paraclinical outcomes.

Methods: We collected data for the patients who underwent a cardiac transplant in our center between 2011 and 2023, forming an initial cohort of 51 patients. Seven patients were excluded for incomplete data sheets, resulting in a final cohort of 44 patients. Means and medians were compared between the ischemic and nonischemic groups, and associations were investigated for each group with hospitalization parameters and clinical and histopathological outcomes.

Results: We found that the patients in the ischemic group had a higher mean age, weight, body mass index, and body surface area, while the nonischemic group has been associated with the development of the Quilty effect and graft rejection. Furthermore, the expression of the human leukocyte antigen-A variant was associated with graft rejection and Quilty effect.

Conclusion: Identifying factors that may predict or contribute to postoperative success is of utmost importance. Further studies are required to validate these results.

## Introduction

Heart failure (HF) is recognized as a clinical syndrome characterized by an inability of the heart to adequately fulfill its role in the cardiovascular system, usually through a reduced pump function or a filling disorder, or secondary to an increased ventricular filling pressure in case of a normal cardiac output (e.g., increased neurohormonal activation in several systemic diseases such as arterial hypertension and obesity) [1]. The main symptoms associated with HF are dyspnea, fatigue, peripheral edema, orthopnea, and bendopnea [2].

The first classification of HF based on the intensity of symptoms was adopted in 1927-1928 under the form of the New York Heart Association (NYHA) classification, to allow physicians to use a common language to communicate their findings and to report on their experience [3]. The NYHA classification divides symptoms into four intensity classes: I: no limitation of activity by symptoms of HF; II: slight limitation of activity, mild symptoms during physical effort; III: marked limitation of activity, less than ordinary physical effort causes symptoms; and IV: symptoms at rest, unable to carry on any physical activity without symptoms.

Over the past decades, several drugs have proven themselves as pillars of treatment in chronic HF, with clear benefits observed not only in improvement of the clinical picture but also in survival and reduced hospitalization rates. These include beta-blockers [4], angiotensin receptor-neprilysin inhibitors [5], angiotensin-converting enzyme inhibitors/angiotensin II receptor blockers [6], and sodium-glucose cotransporter-2 inhibitors [7], together with loop diuretics for decongestion. While new medications continued to reveal their usefulness in treating this syndrome, in certain cases, implantable devices have proven to have unmatched efficacy. In the context of inter- and intraventricular dissynchrony, such as in the presence of a major left bundle branch block, pacemakers used for cardiac resynchronization therapy (CRT) have changed the way in which physicians see the pathophysiology of different phenotypes of HF. Simultaneously, both medical therapies and interventional therapies are constantly developing, with more refined forms of each technique being one of the main subjects in contemporary HF updates [8].

Even though the previously mentioned therapies are constantly improving and refining HF management, the final treatment option for end-stage HF is represented by the heart transplant. Paradoxically, the constant improvements of medical and interventional treatments, while improving the quality of life and life expectancy of HF patients, have the important consequence of increasing the number of patients who will reach an advanced stage of the disease at an elderly age, maintaining a high demand for heart transplants [9-11].

The first description of the procedure dates from 1967, when Christian Barnard performed the first heart transplantation [12]. The surgical technique was developed and refined throughout the years, with impressive survival rates in modern times: 89% survival one year after the procedure, with a median worldwide survival of 10 years [13,14].

Organ availability is one of the most challenging aspects of the matter, as the increase in survivability and slowing of disease progression has also increased the number of patients who will be placed on waiting lists for a heart transplant. Studies have shown that the discrepancy between organ availability and patients requiring transplantation has remained at relatively high levels despite constant efforts to reduce this gap [15,16].

To maximize the results and increase the life expectancy of heart transplant recipients, research has focused on identifying factors that influence the prognosis and management of this fragile category of patients. These include clinical and paraclinical characteristics of patients and their potential link to postoperative complications (e.g., death, graft rejection, vasculitis, and acute kidney injury (AKI)) that might influence both the prognosis and therapeutic algorithm.

This study aimed to analyze the groups of patients who underwent cardiac transplantation in our center and investigate whether the etiology of the underlying cardiomyopathy that was associated with end-stage HF is linked to specific postoperative outcomes and complications. A secondary objective of the study was to evaluate any potential influence of the human leukocyte antigen-A (HLA-A) profiles on the postoperative outcomes.

## Materials and methods

**Figure 1:**
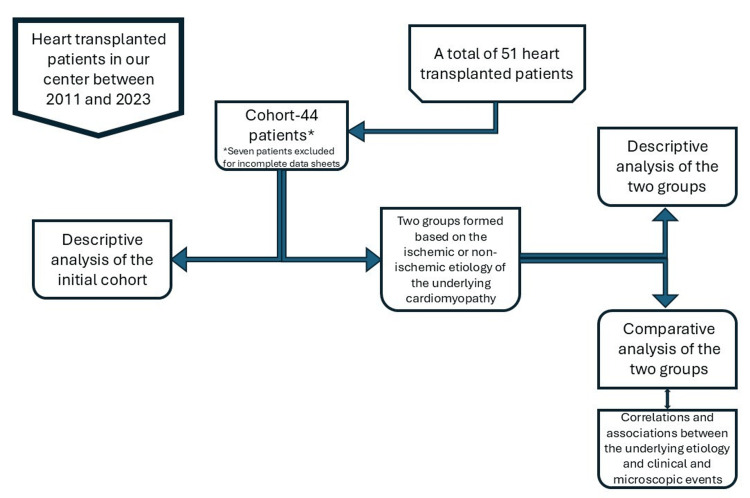
Schematic of the study

The cohort analyzed in our study comprised patients admitted to the Emergency Institute for Cardiovascular Diseases and Transplant of Târgu Mureș for heart transplant between 2011 and 2023. The total number of initially included patients was 51, of whom seven were later excluded due to incomplete data records, resulting in a final cohort of 44 patients (Figure 1).

Informed consent was obtained from the participants before the transplant. We conducted the study in accordance with the Declaration of Helsinki, while the study protocol was approved by the Ethics Committee of the Emergency Institute of Cardiovascular Diseases and Transplant of Târgu Mureș (approval no. 1374/13.02.2024).

The surgical procedure chosen for all participants was the bicaval technique, due to its lower rate of mechanical complications (e.g., mitral or tricuspid regurgitation) and higher rate of preserving postoperative sinus rhythm, secondary to a more physiological structure of the right atrium [17].

Data were collected from the medical sheets for all patients regarding gender, age, weight, body surface area (BSA), body mass index (BMI), etiology of the underlying cardiomyopathy (ischemic or nonischemic), duration of hospital stay, duration of intensive care unit (ICU) stay, time under intravenous (i.v.) inotropes or vasopressors, number of inotropes or vasopressors required for the patient, history of a cardiac implantable device (implantable cardioverter-defibrillator (ICD), cardiac resynchronization therapy pacemaker or cardiac resynchronization therapy pacemaker with defibrillator) implantation, six-month survival, postoperative clinical complications (e.g., atrial fibrillation (AF), AKI, persistent hyperglycemia requiring subcutaneous (s.c.), or i.v. correction with insulin of newly onset diabetes mellitus (DM)), and histopathological events seen on endocardial biopsies during the admission in which the surgery was performed (e.g., any stage of graft rejection, vasculitis, and presence of the Quilty effect). Survival and follow-up results were assessed from the Intranet system of the Emergency Institute of Cardiovascular Diseases and Transplant of Târgu Mureș, and descriptive statistics for the baseline characteristics of the cohort were performed.

To fulfill the aim of this paper, we further divided our cohort into two groups with respect to the ischemic or nonischemic etiology of the underlying cardiomyopathy that led to end-stage HF. To analyze parameters consisting of binary data, associations were investigated between the etiology of the cardiomyopathy and gender, previous cardiac implantable devices, six-month survival, AF, AKI, DM, graft rejection, vasculitis, and Quilty effect observed on the histopathological analysis of the biopsy probes of the recipients. For continuous data, means and medians are reported for age, weight, BSA, BMI, duration of hospital and ICU stay, as well as the number and duration of i.v. Inotrope treatment was compared using the corresponding parametric or nonparametric tests. The Quilty effect is defined as large endocardial infiltrates seen after cardiac transplantation. The HLA-A has been analyzed for associations with the aforementioned outcomes, from the data sheets that contained a complete record of the recipient’s profile.

IBM Statistical Package for the Social Sciences Statistics version 30.0.0 (IBM Corp., Armonk, NY) was the platform used to perform all statistical analyses. Means, medians, standard deviations (SDs), maximum, and minimum were determined for quantitative data. Assessment of the normal distribution of values was performed using the Shapiro-Wilk test. We considered a statistical threshold of 0.05 for all statistical tests.

## Results

First, baseline characteristics of the patients in the primary cohort were extracted. Thus, out of the 44 patients, 38 were male patients (86.36%), and six were female patients (13.64%). The mean age was 41.84 years (SD = 13.49), and the mean weight was 71.72 kg (SD = 19.07). The mean BMI was 23.59 kg/m^2^ (SD = 4.74), while the mean BSA was 1.84 m^2^ (SD = 0.30). Ten patients (22.72%) had an ischemic etiology of the underlying cardiopathy, while 34 individuals (77.28%) presented a nonischemic etiology of the disease. Regarding admission time, the mean hospitalization time was 60.72 days (SD = 65.45), with a mean duration of ICU stay of 52.02 days (SD = 62.12). The mean duration of i.v. inotropic/vasopressor treatment was 6.65 days (SD = 6.77) with a mean number of 2.40 agents per patient (SD = 1.08). Out of the 44 patients, 24 (54.54%) had a previously implanted cardiac device (ICD or CRT), while 20 did not receive such a device before the transplant (45.46%). Only two patients (4.54%) did not survive past the six-month threshold, while 42 (95.46%) were still alive after six months.

Second, both clinical and histopathological complications that occurred after the heart transplant were assessed. After the procedure, six patients (13.63%) developed AF, while 38 (86.37%) maintained sinus rhythm. Twenty-three patients (52.27%) developed AKI, and 29 individuals (65.90%) had hyperglycemia requiring i.v. or s.c. insulin administration. Regarding microscopic events highlighted on myocardial biopsies, 11 patients (25%) experienced changes suggestive of graft rejection, while biopsies from 30 patients (68.18%) showed changes suggestive of vasculitis. The Quilty effect was present on specimens collected from 14 patients (31.81%) (Tables 1, 2).

**Table 1:** Central tendencies of baseline characteristics for the initial cohort SD, standard deviation; BMI, body mass index; BSA, body surface area; ICU, intensive care unit

Parameter	Mean	Median	SD
Age, years	41.84	46.00	13.49
Weight, kg	71.72	71.50	19.07
BMI, kg/m^2^	23.59	23.95	4.74
BSA, m^2^	1.84	1.88	0.30
Hospitalization, days	60.72	39.00	65.45
ICU stay, days	52.02	34.50	62.16
Duration of inotrope treatment, days	6.65	4.00	6.77
Number of inotropes, n	2.40	2.00	1.08

**Table 2:** General characteristics of the initial cohort

Parameter/event	Present, n (%)	Absent, n (%)
Gender, male	38 (86.36)	6 (13.64)
Etiology, ischemic	10 (22.72)	34 (77.28)
Six-month survival, survived	42 (95.40)	2 (4.60)
Implantable device, yes	24 (54.55)	20 (45.45)
Atrial fibrillation, yes	6 (13.64)	38 (86.36)
Acute kidney injury, yes	23 (52.27)	21 (47.73)
Diabetes mellitus or uncontrolled hyperglycemia	29 (65.90)	15 (34.10)
Graft rejection, yes	11 (25.00)	33 (75.00)
Vasculitis, yes	30 (68.18)	14 (31.82)
Quilty effect, yes	14 (31.82)	30 (68.18)

The following stage of the study included the formation of subgroups regarding the ischemic or nonischemic etiology of the underlying cardiomyopathy and characterization of these populations. Thus, the ischemic group consisted of 10 patients, with a 100% (n = 10) proportion of males. The mean age in this group was 52.10 years (SD = 6.95), while the mean weight was 85.80 kg (SD = 13.49), the mean BMI was 28.02 kg/m^2^ (SD = 3.42), and the mean BSA was 2.03 m^2^ (SD = 0.18). The mean hospitalization duration for the ischemic group was 38.60 days (SD = 12.03), while the mean ICU stay was 34.50 days (SD = 13.41). The mean duration of inotropic or vasopressor treatment was 3.60 days (SD = 1.42), with a mean of 1.70 agents (SD = 0.82) required to maintain an appropriate blood pressure. Of the 10 patients in this group, six (60%) have previously received an implantable device (ICD or CRT). All 10 patients (100%) survived past the six-month threshold. Regarding clinical and histopathological complications, none of the patients (0%) developed AF postoperatively, five patients (50%) developed AKI, and eight patients (80%) developed DM or uncontrolled hyperglycemia. None of the patients (0%) developed microscopic changes suggestive of graft rejection; six (60%) presented features suggestive of vasculitis; and none (0%) showed the Quilty effect on cardiac biopsies (Tables 3, 4).

**Table 3:** Central tendencies of baseline characteristics for the ischemic group SD, standard deviation; BMI, body mass index; BSA, body surface area; ICU, intensive care unit

Parameter	Mean	Median	SD
Age, years	52.10	51.50	6.95
Weight, kg	85.80	86.00	13.49
BMI, kg/m^2^	28.02	28.85	3.42
BSA, m^2^	2.03	2.02	0.18
Hospitalization, days	38.60	37.00	12.03
ICU stay, days	34.50	32.00	13.41
Duration of inotrope treatment, days	3.60	4.00	1.42
Number of inotropes, n	1.70	1.50	0.82

**Table 4:** General characteristics of the two groups

Parameter/event	Ischemic group (n = 10), n (%)	Nonischemic group (n = 34), n (%)
Gender, male	10 (100)	28 (82.35)
Six-month survival, survived	10 (100)	32 (94.11)
Implantable device, yes	6 (60)	18 (52.94)
Atrial fibrillation, yes	0 (0)	6 (17.64)
Acute kidney injury, yes	5 (50)	18 (52.94)
Diabetes mellitus or uncontrolled hyperglycemia	8 (80)	21 (61.76)
Graft rejection, yes	0 (0)	11 (32.35)
Vasculitis, yes	6 (60)	24 (70.58)
Quilty effect, yes	0 (0)	14 (41.17)

The following parameters characterized the nonischemic group: a total of 34 patients, consisting of 28 male patients (82.35%) and six female patients (17.65%), with a mean age of 38.82 years (SD = 13.52) and a mean weight of 67.58 kg (SD = 18.62). This group was characterized by a mean BMI of 22.29 kg/m^2^ (SD = 4.29) and a mean BSA of 1.79 m^2^ (SD = 0.31). The mean hospitalization duration was 67.23 days (SD = 73.15), with a mean ICU stay of 57.17 days (SD = 69.75). The mean duration of inotropic support was 7.55 days (SD = 7.45), and a mean of 2.61 inotropic agents (SD = 1.07) were necessary to maintain appropriate perfusion. Of the 34 patients in this group, 18 (52.94%) had a previously implanted cardiac device (ICD or CRT) at the time of surgery. Thirty-two of the patients (94.11%) survived past the six-month threshold, while two (5.89%) did not reach this milestone. The analysis of clinical complications showcased six patients (17.64%) who developed AF, while 28 (82.36%) maintained sinus rhythm after the transplant. Eighteen patients (52.94%) developed AKI, and 21 (61.76%) presented either newly onset DM or uncontrolled hyperglycemia. When examining the biopsies, 11 patients (32.35%) developed changes suggestive of graft rejection, 24 (70.58%) developed signs of vasculitis, and 14 (41.17%) showed the Quilty effect (Tables 4, 5).

**Table 5:** Central tendencies of baseline characteristics for the nonischemic group SD, standard deviation; BMI, body mass index; BSA, body surface area; ICU, intensive care unit

Parameter	Mean	Median	SD
Age, years	38.82	41.00	13.52
Weight, kg	67.58	68.00	18.62
BMI, kg/m^2^	22.29	22.60	4.29
BSA, m^2^	1.79	1.81	0.31
Hospitalization, days	67.23	41.50	73.15
ICU stay, days	57.17	35.00	69.75
Duration of inotrope treatment, days	7.55	5.00	7.45
Number of inotropes, n	2.61	2.50	1.07

To investigate any potentially significant differences between the groups, concerning the etiology of the cardiomyopathy, central tendencies were compared for the continuous data, and contingency tables were used to probe potential associations between binary data and the etiology of the cardiomyopathy. Thus, central tendencies were compared for age (p < 0.01), weight (p < 0.01), BMI (p < 0.01), BSA (p = 0.02), hospitalization time (p = 0.16), duration of ICU stay (p = 0.19), duration of inotropic/vasopressor treatment (p = 0.07), and the number of needed inotropes (p = 0.01). For associations, we used contingency tables to compare the nonischemic and ischemic groups for male gender (odds ratio, OR = 0.20, 95% confidence interval, CI = 0.01-4.03, p = 0.30), six-month survival (OR = 0.61, 95% CI = 0.02-13.95, p = 0.76), previously implanted device (OR = 0.75, 95% CI = 0.17-3.14, p = 0.69), and postoperative clinical complications under the form of AF (OR = 4.78, 95% CI = 0.24-92.63, p = 0.30), AKI (OR = 1.12, 95% CI = 0.27-4.61, p = 0.87) and DM (OR = 0.40, 95% CI = 0.07-2.20, p = 0.29), as well as complications observed on the cardiac biopsies, such as changes suggestive for any stage of graft rejection (OR = 1.47, 95% CI = 1.17-1.86, p = 0.04), vasculitis (OR = 1.6, 95% CI = 0.37-6.92, p = 0.70), and Quilty effect (OR = 1.70, 95% CI = 1.28-2.25, p = 0.18) (Tables 6, 7).

**Table 6:** Results for central tendencies BMI, body mass index; BSA, body surface area; ICU, intensive care unit

Parameter	Performed test	p value	Significance
Age	Mann-Whitney test	<0.01	Yes
Weight	Student's t-test for equal variances	<0.01	Yes
BMI	Student's t-test for equal variances	<0.01	Yes
BSA	Student's t-test for equal variances	0.02	Yes
Hospitalization	Mann-Whitney test	0.16	No
ICU stay	Mann-Whitney test	0.19	No
Time under inotropes	Mann-Whitney test	0.07	No
Number of inotropes	Mann-Whitney test	0.01	Yes

**Table 7:** Results for events and binary data ^*^Statistically significant

Parameter	Odds ratio	Confidence interval	Significance
Gender, male	0.20	0.01-4.03	0.30
Six-month survival, survived	0.61	0.02-13.95	0.76
Implantable device, yes	0.75	0.17-3.14	0.69
Atrial fibrillation, yes	4.78	0.24-92.63	0.30
Acute kidney injury, yes	1.12	0.27-4.61	0.87
Diabetes mellitus or uncontrolled hyperglycemia, yes	0.40	0.07-2.20	0.29
Graft rejection, yes	1.47	1.17-1.86	0.04^*^
Vasculitis, yes	1.60	0.37-6.92	0.70
Quilty effect, yes	1.70	1.28=2.25	0.01^*^

The profile of the subgroup of patients with complete HLA-A expression records has been analyzed (Table 8).

**Table 8:** HLA-A profile of the patients HLA-A, human leukocyte antigen-A

Profile	Profile	Profile	Profile
A25, A30	A24, A24	A24, A24	A2, A24
A2, A11	A11, A68	A1, A11	A2, A68
A2, A26	A1, A2	A2, A30	A1, A68
A1, A2	A1, A24	A29, A31	A2, A2
A2, A24	A1, A32	A11, A24	A26, A68
A1, A2	A31, A32	A1, A2	A2, A2
A1, A2	A2, A66	A1, A3	A2, A24
A31, A68	A2, A2	A11, A24	A24, A24

Associations between each genotype and postoperative outcomes were investigated: six-month survival (A1, p = 0.53; A2, p =1.00; A3, p = 1.00; A11, p = 0.29; A24, p = 0.44; A25, p = 1.00; A26, p = 1.00; A29, p = 1.00; A30, p = 1.00; A31, p = 1.00; A32, p = 1.00; A66, p = 1.00; A68, p = 0.61), AF (A1, p = 0.15; A2, p = 1.00; A3, p = 1.00; A11, p = 0.56; A24, p = 0.57; A25, p = 1.00; A26, p = 0.29; A29, p = 1.00; A30, p = 1.00; A31, p = 0.41; A32, p = 0.29; A66, p = 1.00; A68, p = 0.16), AKI (A1, p = 1.00; A2, p = 1.00; A3, p = 1.00; A11, p = 0.63; A24, p = 0.41; A25, p = 0.43; A26, p = 1.00; A29, p = 1.00; A30, p = 0.18; A31, p = 1.00; A32, p = 1.00; A66, p = 1.00; A68, p = 1.00), DM (A1, p = 0.43; A2, p = 0.71; A3, p = 1.00; A11, p = 1.00; A24, p = 1.00; A25, p = 1.00; A26, p = 0.51; A29, p = 1.00; A30, p = 0.51; A31, p = 1.00; A32, p = 1.00; A66, p = 1.00; A68, p = 1.00), graft rejection (A1, p = 1.00; A2, p = 1.00; A3, p = 1.00; A11, p = 0.60; A24, p = 1.00; A25, p = 1.00; A26, p = 0.49; A29, p = 1.00; A30, p = 1.00; A31, p = 1.00; A32, p = 1.00; A66, p = 1.00; A68, p = 0.01^*^, OR = 17.60, CI = 1.60-193.40), vasculitis (A1, p = 0.40; A2, p = 1.00; A3, p = 0.28; A11, p = 1.00; A24, p = 1.00; A25, p = 0.28; A26, p = 0.49; A29, p = 0.28; A30, p = 0.49; A31, p = 1.00; A32, p = 1.00; A66, p = 1.00; A68, p = 0.28), the Quilty effect (A1, p = 1.00; A2, p = 0.70; A3, p = 1.00; A11, p = 0.29; A24, p = 0.68; A25, p = 1.00; A26, p = 0.53; A29, p = 1.00; A30, p = 1.00; A31, p = 1.00; A32, p = 1.00; A66, p = 1.00; A68, p = 0.02^*^, OR = 14.00, 95% CI = 1.36-144.50) (Tables 9, 10).

**Table 9:** Associations between HLA-A expression and clinical complications HLA-A, human leukocyte antigen-A; AF, atrial fibrillation; AKI, acute kidney injury; DM, diabetes mellitus

HLA-A	Six-month survival	AF	AKI	DM
A1	0.53	0.15	1.00	0.43
A2	1.00	1.00	1.00	0.71
A3	1.00	1.00	1.00	1.00
A11	0.29	0.56	0.63	1.00
A24	0.44	0.57	0.41	1.00
A25	1.00	1.00	0.43	1.00
A26	1.00	0.29	1.00	0.51
A29	1.00	1.00	1.00	1.00
A30	1.00	1.00	0.18	0.51
A31	1.00	0.41	1.00	1.00
A32	1.00	0.29	1.00	1.00
A66	1.00	1.00	1.00	1.00
A68	0.61	0.16	1.00	1.00

**Table 10:** Associations between HLA-A expression and pathological complications HLA-A, human leukocyte antigen-A ^*^Statistically significant

HLA-A	Graft rejection	Vasculitis	Quilty effect
A1	1.00	0.40	1.00
A2	1.00	1.00	0.70
A3	1.00	0.28	1.00
A11	0.60	1.00	0.29
A24	1.00	1.00	0.68
A25	1.00	0.28	1.00
A26	0.49	0.49	0.53
A29	1.00	0.28	1.00
A30	1.00	0.49	1.00
A31	1.00	1.00	1.00
A32	1.00	1.00	1.00
A66	1.00	1.00	1.00
A68	0.01^*^	0.28	0.02^*^

## Discussion

As treatment for HF is constantly improving, with slower disease progression, the number of patients who will reach end-stage HF at an older age will paradoxically increase, due to the prolongation of the early stages of the disease. This tendency has several notable disadvantages, the first of which is the relatively low availability of donated hearts, highlighting the need for thorough selection and matching of donor and recipient, as well as identifying factors that may predict short- and long-term outcomes in the context of a widening discrepancy between donated and required organs [18]. Another challenge in the management of older patients with HF is the relative reluctance of physicians to uptitrate medication to the target dose due to frailty concerns, making it more challenging to manage these patients [19].

Once all other means of treatment have been exhausted and the indication for heart transplantation has been made, a suitable donor must be selected. Canonically, an exact match is required to perform a heart transplantation. Due to the previously mentioned scarcity of donated organs, new directions have to be explored, both to expand the pool of organs through imperfect matching or xenograft transplantation [20] and to improve postoperative management, both through medication and prevention of complications. As identifying patients’ characteristics that may predict complications or raise awareness of potential negative outcomes becomes more important, studies are needed to fulfill this objective. In our research, we retrospectively analyzed the data of patients who underwent cardiac transplantation during 12 years with the focus of identifying factors suggestive of postoperative complications and short- and mid-term outcomes.

In light of the previously mentioned aim, we determined the general characteristics of the cohort and noted that most of the patients who underwent heart transplant in our center were male patients, with a mean BMI in the normosthenic range. The pathologies of nonischemic origin represented the majority of underlying cardiomyopathies connected to the recipients’ end-stage disease. These data are consistent with previously reported information from international multiannual databases [21].

Differences in outcomes about patients’ characteristics are one of the focal points of nowadays’ transplant research, highlighting the importance of individual factors that could influence the following therapeutic algorithm. To this day, the importance of gender, age, and coexisting comorbidities has been studied, and their status has been part of acute management and postoperative follow-up, with a focus on the potential complications that may occur more frequently in specific cases [21-23].

To investigate any potential correlations or associations between the etiology of the underlying cardiomyopathy and specific postoperative outcomes, the cohort was divided into two groups regarding the underlying disease. In terms of baseline characteristics, the ischemic group was defined by significantly higher median age, mean weight, mean BMI, and mean BSA, which is confirmed by the progressive nature of ischemic disease, atheromatous lesions being naturally associated with higher age and weight. There was no statistically significant difference between hospitalization time, time in the ICU, and time under inotrope/vasopressor treatment, but there was a significant difference in the number of inotropes needed to support postoperative homeostasis, with the nonischemic group requiring more agents.

Regarding postoperative complications, there was a statistically significant association between the nonischemic etiology of the underlying cardiomyopathy and graft rejection, as well as with the Quilty effect. These findings could have important implications, as nonischemic etiologies sometimes have systemic causes or may be part of a systemic syndrome [24].

Comparing our findings with the available literature, Szymanska et al. showed a relation between the Quilty effect and graft rejection [25], while very little data are available on the relation between the Quilty effect and the etiology of the recipient’s cardiomyopathy. The nonsignificant difference in short- and mid-term survival between the ischemic and nonischemic recipients of a heart transplant revealed by our study was confirmed by other studies [26]. Given the lack of recent literature on the relationship between the ischemic or nonischemic characteristics of the recipient, it is necessary to consider the results of smaller studies, which may indicate areas for future research. The overall status of the recipient’s organism differs greatly when considering an ischemic disease with increased inflammation and potential peripheral lesions, or a patient with a systemic condition related to genetic diseases, which may affect multiple organs. This raises awareness of the need for individualized management of heart-transplant patients.

These histopathological changes have been linked to graft rejection [27], with an unclear role in other complications such as postoperative coronary vasculopathy, which has also been linked to improved survival [28]. As technology advances, the Quilty effect has once again been addressed as a research area, with a statement at the XVth Banff Conference (2020) to reassess the Quilty effect at the microscopic and molecular levels in the future [29].

**Figure 2:**
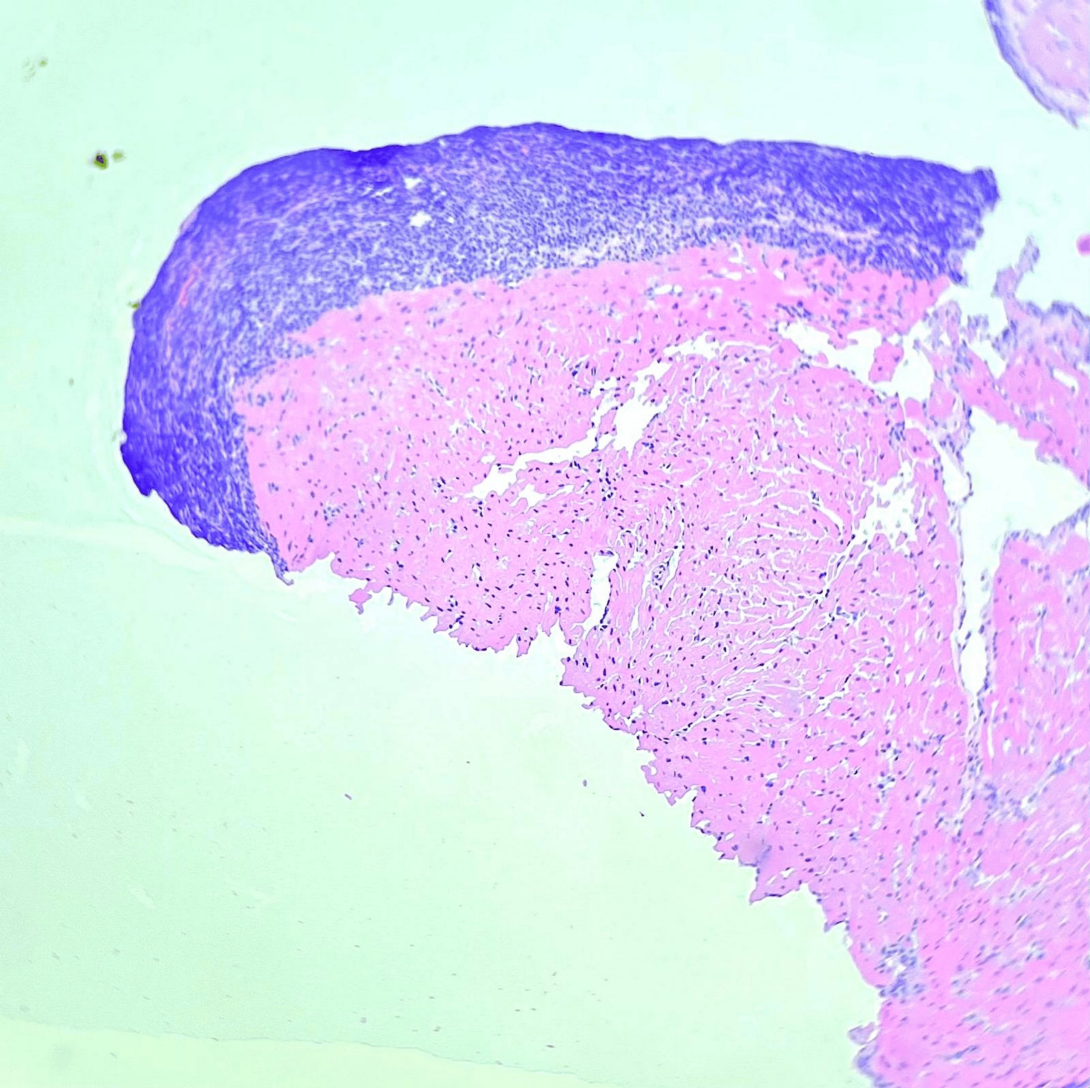
The Quilty effect on samples taken out of endomyocardial biopsies (20× magnification)

**Figure 3:**
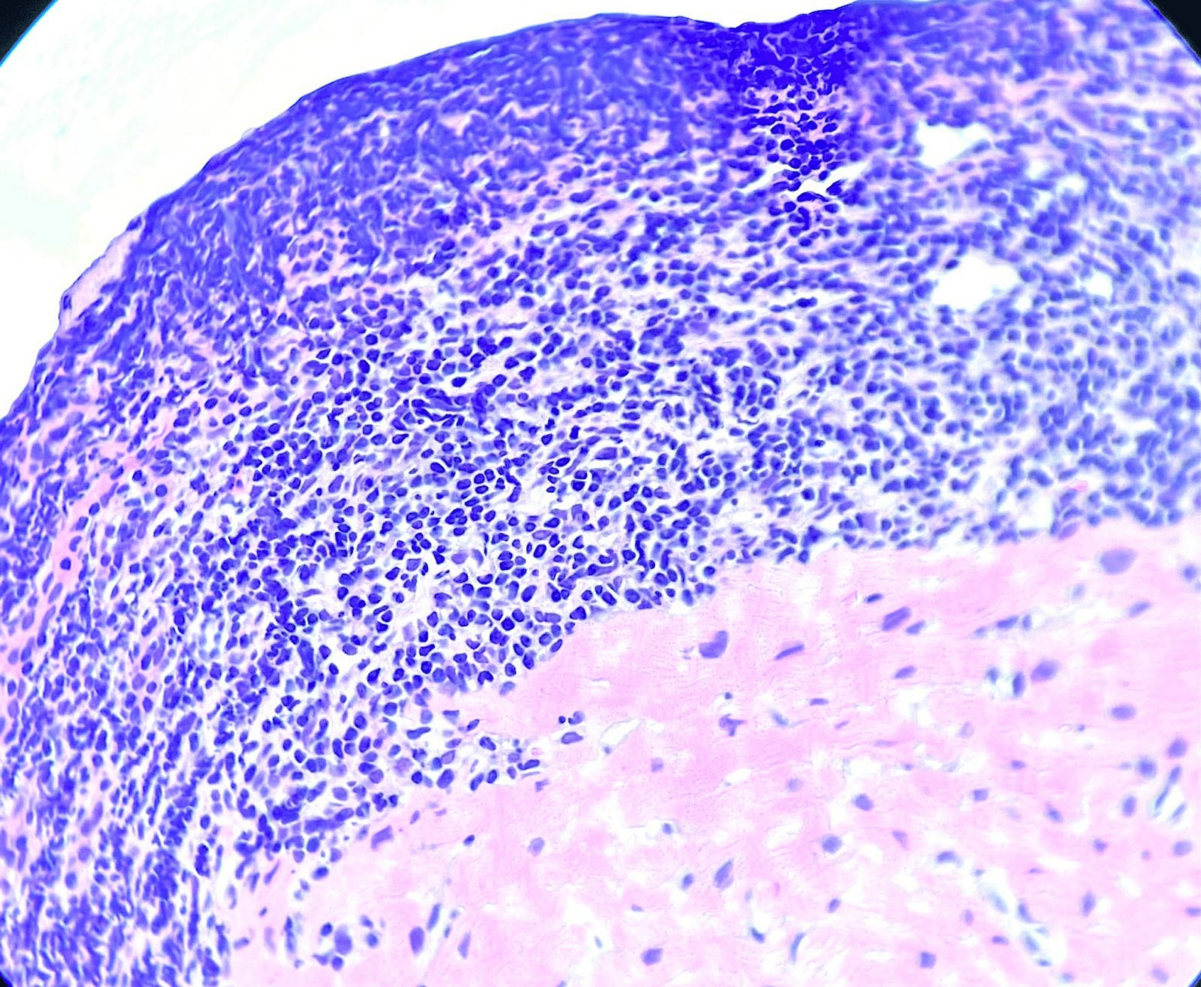
The Quilty effect on samples taken out of endomyocardial biopsies (40× magnification)

In the second part of our research, the subgroup of patients who had complete data records for their HLA-A genotype was investigated. Thus, we found a significant association between the presence of a heterozygous HLA-A68 variant and graft rejection, as well as the Quilty effect (Figures 2, 3). This may prove to be important in the future selection and management of heart transplantation recipients. Available literature describes the HLA-A68 variant as highly polymorphic, which may increase alloreactivity and enhance antibody-mediated processes, potentially leading to more graft rejections. The polymorphism of this gene is the cause of inferior allograft survival in cases of mismatch [30-32].

Thus, identifying factors that predict these pathological changes is of utmost importance and should play a role in the management of patients who undergo a heart transplant, once larger prospective studies validate the findings. The limitations of studies focused on organ transplantation must be acknowledged, as the fragile nature of these patients, alongside the multisystemic approach for their management and the social implications, make prospective research on this topic very scarce.

First, there is an increasing gap between the required organs for transplant and available donors, despite recent efforts from medical communities to raise awareness on this topic. In Eastern European countries, this issue is even more pressing, as religious beliefs and lack of legislation prove to be important obstacles in convincing people of the impact and benefits of organ transplantation [33]. Romania, in particular, is one of the countries gravely affected by the legislative gap regarding organ donation, as to become a donor, an individual must personally submit the application to the organ donor registry, or consent must be obtained from a close relative upon death. As awareness campaigns are not widely popular, most of the cases of organ donation happen in the latter scenario, forcing grieving relatives to make the important decision of estranging parts of a loved one in the early moments after their loss [34].

The psychological impact on close relatives is not isolated to the donor’s family, as caring for an end-stage HF patient has a tremendous psychological impact on the future recipient’s family, some studies showcasing signs of depression in these individuals, as well as a decreased quality of life [35]. Thus, increasing the availability of organs through media campaigns, education, and legislation becomes of utmost importance for many individuals.

Limitations of the study must be acknowledged, though they are tributary to the previously mentioned challenges in heart transplant management in the country. The small number of patients included may be a source of false-positive results, and the retrospective character of the study makes it less reliable for providing solid medical evidence. Nonetheless, future research in this area is needed, and small retrospective studies may be the starting point of larger, prospective research [36].

## Conclusions

In this 12-year retrospective analysis of heart transplant recipients, nonischemic cardiomyopathy and the presence of a heterozygous HLA-A68 genotype were associated with higher rates of graft rejection and the Quilty effect. These findings highlight the importance of incorporating both the etiology of end-stage cardiomyopathy and immunogenetic profiling into posttransplant surveillance strategies. Larger, prospective studies are needed to further validate these findings.

## References

[1] Savarese G, Becher PM, Lund LH, Seferovic P, Rosano GM, Coats AJ (2023). Global burden of heart failure: a comprehensive and updated review of epidemiology. Cardiovasc Res.

[2] Chen J, Aronowitz P (2022). Congestive heart failure. Med Clin North Am.

[3] Caraballo C, Desai NR, Mulder H (2019). Clinical implications of the New York Heart Association classification. J Am Heart Assoc.

[4] Prijic S, Buchhorn R (2014). Mechanisms of beta-blockers action in patients with heart failure. Rev Recent Clin Trials.

[5] Tromp J, Ouwerkerk W, van Veldhuisen DJ (2022). A systematic review and network meta-analysis of pharmacological treatment of heart failure with reduced ejection fraction. JACC Heart Fail.

[6] Tomasoni D, Adamo M, Lombardi CM, Metra M (2019). Highlights in heart failure. ESC Heart Fail.

[7] Beghini A, Sammartino AM, Papp Z (2025). 2024 update in heart failure. ESC Heart Fail.

[8] McDonagh TA, Metra M, Adamo M (2021). 2021 ESC guidelines for the diagnosis and treatment of acute and chronic heart failure. Eur Heart J.

[9] Habal MV, Garan AR (2017). Long-term management of end-stage heart failure. Best Pract Res Clin Anaesthesiol.

[10] Levy WC, Mozaffarian D, Linker DT (2006). The Seattle Heart Failure Model: prediction of survival in heart failure. Circulation.

[11] Allen LA, Stevenson LW, Grady KL (2012). Decision making in advanced heart failure: a scientific statement from the American Heart Association. Circulation.

[12] Awad MA, Shah A, Griffith BP (2022). Current status and outcomes in heart transplantation: a narrative review. Rev Cardiovasc Med.

[13] Hsich EM, Blackstone EH, Thuita LW (2020). Heart transplantation: an in-depth survival analysis. JACC Heart Fail.

[14] Giblin G, Murphy L, Mahon N (2020). Survival trends post cardiac transplantation: a comparative analysis of Irish and international data (1985-2019). J. Heart Lung Transplant.

[15] Hart A, Lentine KL, Smith JM (2021). OPTN/SRTR 2019 annual data report: kidney. Am J Transplant.

[16] Dharmavaram N, Hess T, Jaeger H, Smith J, Hermsen J, Murray D, Dhingra R (2021). National trends in heart donor usage rates: are we efficiently transplanting more hearts?. J Am Heart Assoc.

[17] Toscano G, Bottio T, Gambino A (2015). Orthotopic heart transplantation: the bicaval technique. Multimed Man Cardiothorac Surg.

[18] Arriola K, Robinson D (2009). Narrowing the gap between supply and demand of organs for transplantation. Health Issues in the Black Community.

[19] Veenis JF, Brunner-La Rocca HP, Linssen GC (2019). Age differences in contemporary treatment of patients with chronic heart failure and reduced ejection fraction. Eur J Prev Cardiol.

[20] Zachary AA, Leffell MS (2016). HLA mismatching strategies for solid organ transplantation - a balancing act. Front Immunol.

[21] Hickey KT, Doering LV, Chen B (2017). Clinical and gender differences in heart transplant recipients in the NEW HEART study. Eur J Cardiovasc Nurs.

[22] Tsao CW, Aday AW, Almarzooq ZI (2023). Heart disease and stroke statistics-2023 update: a report from the American Heart Association. Circulation.

[23] Frankenstein L, Clark AL, Ribeiro JP (2012). Influence of sex on treatment and outcome in chronic heart failure. Cardiovasc Ther.

[24] Arbelo E, Protonotarios A, Gimeno JR (2023). 2023 ESC guidelines for the management of cardiomyopathies. Eur Heart J.

[25] Szymanska S, Grajkowska W, Sobieszczanska-Malek M, Zielinski T, Pyzlak M, Pronicki M (2016). Prevalence of the Quilty effect in endomyocardial biopsy of patients after heart transplantation - from cellular rejection to antibody-mediated rejection?. Pol J Pathol.

[26] Oehler D, Bruno RR, Holst HT (2022). Ischemic versus nonischemic recipient indication does not impact outcome after heart transplantation. Exp Clin Transplant.

[27] Zakliczynski M, Nozynski J, Konecka-Mrowka D (2009). Quilty effect correlates with biopsy-proven acute cellular rejection but does not predict transplanted heart coronary artery vasculopathy. J Heart Lung Transplant.

[28] Chu KE, Ho EK, de la Torre L, Vasilescu ER, Marboe CC (2005). The relationship of nodular endocardial infiltrates (Quilty lesions) to survival, patient age, anti-HLA antibodies, and coronary artery disease following heart transplantation. Cardiovasc Pathol.

[29] Duong Van Huyen JP, Fedrigo M, Fishbein GA (2020). The XVth Banff Conference on Allograft Pathology the Banff Workshop Heart Report: improving the diagnostic yield from endomyocardial biopsies and Quilty effect revisited. Am J Transplant.

[30] DiSesa VJ, Kuo PC, Horvath KA, Mudge GH, Collins JJ Jr, Cohn LH (1990). HLA histocompatibility affects cardiac transplant rejection and may provide one basis for organ allocation. Ann Thorac Surg.

[31] Chen CK, Manlhiot C, Conway J, Allain-Rooney T, McCrindle BW, Tinckam K, Dipchand AI (2015). Development and impact of de novo anti-HLA antibodies in pediatric heart transplant recipients. Am J Transplant.

[32] Santos E, Spensley K, Gunby N, Worthington J, Roufosse C, Anand A, Willicombe M (2024). Application of HLA molecular level mismatching in ethnically diverse kidney transplant recipients receiving a steroid-sparing immunosuppression protocol. Am J Transplant.

[33] Lewis A, Koukoura A, Tsianos GI, Gargavanis AA, Nielsen AA, Vassiliadis E (2021). Organ donation in the US and Europe: the supply vs demand imbalance. Transplant Rev (Orlando).

[34] Holman A, Karner-Huţuleac A, Ioan B (2013). Factors of the willingness to consent to the donation of a deceased family member's organs among the Romanian urban population. Transplant Proc.

[35] Burker EJ, Evon DM, Ascari JC, Loiselle MM, Finkel JB, Mill MR (2006). Relationship between coping and depression in heart transplant candidates and their spouses. Prog Transplant.

[36] Hackshaw A (2008). Small studies: strengths and limitations. Eur Respir J.

