# Correction: Preoperative Indicators of Graft Rejection in Patients With Heart Transplantation: A Single-Center Retrospective Study

**DOI:** 10.7759/cureus.c407

**Published:** 2026-02-25

**Authors:** Laurentiu Huma, Horatiu Suciu, Diana Andreea Moldovan, Radu-Adrian Suteu, Sergiu Morosanu, Dragos-Florin Baba, Anca-Ileana Sin

**Affiliations:** 1 Department of Doctoral Studies, George Emil Palade University of Medicine, Pharmacy, Science and Technology of Târgu Mureș, Târgu Mureș, ROU; 2 Department of Cell and Molecular Biology, George Emil Palade University of Medicine, Pharmacy, Science and Technology of Târgu Mureș, Târgu Mureș, ROU; 3 Department of Cardiology I, Emergency Institute for Cardiovascular Diseases and Transplantation of Târgu Mureș, Târgu Mureș, ROU; 4 Department of Surgery, George Emil Palade University of Medicine, Pharmacy, Science and Technology of Târgu Mureş, Târgu Mureș, ROU; 5 Department of Cardiovascular Surgery, Emergency Institute for Cardiovascular Diseases and Transplantation of Târgu Mureş, Târgu Mureș, ROU; 6 Department of Family Medicine, George Emil Palade University of Medicine, Pharmacy, Science and Technology of Târgu Mureș, Târgu Mureș, ROU

This article has been corrected to revise the affiliations of several authors.

